# Deployment of Early Diagnosis and Mefloquine- Artesunate Treatment of Falciparum Malaria in Thailand: The Tak Malaria Initiative

**DOI:** 10.1371/journal.pmed.0030183

**Published:** 2006-06-06

**Authors:** Verena Ilona Carrara, Supakit Sirilak, Janjira Thonglairuam, Chaiporn Rojanawatsirivet, Stephane Proux, Valery Gilbos, Al Brockman, Elizabeth A Ashley, Rose McGready, Srivicha Krudsood, Somjai Leemingsawat, Sornchai Looareesuwan, Pratap Singhasivanon, Nicholas White, François Nosten

**Affiliations:** 1Shoklo Malaria Research Unit, Tak, Thailand,; **2**Faculty of Tropical Medicine, Mahidol University, Bangkok, Thailand,; **3**Departments of Medical Entomology, Tropical Hygiene and Clinical Tropical Medicine, Faculty of Tropical Medicine, Mahidol University, Bangkok, Thailand; **4**Public Health Office, Tak, Thailand,; **5**Ministry of Public Health, Nonthaburi, Thailand,; **6**Centre for Vaccinology and Tropical Medicine, Churchill Hospital, Oxford, United Kingdom; Kenya Medical Research InstituteKenya

## Abstract

**Background:**

Early diagnosis and treatment with artesunate-mefloquine combination therapy (MAS) have reduced the transmission of falciparum malaria dramatically and halted the progression of mefloquine resistance in camps for displaced persons along the Thai-Burmese border, an area of low and seasonal transmission of multidrug-resistant Plasmodium falciparum. We extended the same combination drug strategy to all other communities (estimated population 450,000) living in five border districts of Tak province in northwestern Thailand.

**Methods and Findings:**

Existing health structures were reinforced. Village volunteers were trained to use rapid diagnostic tests and to treat positive cases with MAS. Cases of malaria, hospitalizations, and malaria-related deaths were recorded in the 6 y before, during, and after the Tak Malaria Initiative (TMI) intervention. Cross-sectional surveys were conducted before and during the TMI period. P. falciparum malaria cases fell by 34% (95% confidence interval [CI], 33.5–34.4) and hospitalisations for falciparum malaria fell by 39% (95% CI, 37.0–39.9) during the TMI period, while hospitalisations for P. vivax malaria remained constant. There were 32 deaths attributed to malaria during, and 22 after the TMI, a 51.5% (95% CI, 39.0–63.9) reduction compared to the average of the previous 3 y. Cross-sectional surveys indicated that P. vivax had become the predominant species in Thai villages, but not in populations living on the Myanmar side of the border. In the displaced persons population, where the original deployment took place 7 y before the TMI, the transmission of P. falciparum continued to be suppressed, the incidence of falciparum malaria remained low, and the in vivo efficacy of the 3-d MAS remained high.

**Conclusions:**

In the remote malarious north western border area of Thailand, the early detection of malaria by trained village volunteers, using rapid diagnostic tests and treatment with mefloquine-artesunate was feasible and reduced the morbidity and mortality of multidrug-resistant P. falciparum.

## Abstract 2

Artesunate-mefloquine therapy and rapid diagnosis were introduced in five border districts in northwestern Thailand. Cases, hospitalizations, and malaria-related deaths were recorded before, during, and after. Reductions were achieved in morbidity and mortality from multidrug-resistant *P. falciparum.*

## Introduction

The development and spread of multidrug-resistant Plasmodium falciparum parasites has impaired decades of efforts to reduce the burden of malaria worldwide. In Thailand, where the most resistant parasites are found, the National Malaria Control Programme has successfully reduced malaria morbidity and mortality in most of the country using vector control measures and a large network of malaria clinics delivering free diagnosis and treatment [1]. Malaria transmission is now limited to the forested and hilly areas of the Eastern and Western border provinces and the Southern peninsula. The most affected populations are the ethnic minorities, displaced persons, and migrant workers, and the highest incidence is in Tak Province bordering Myanmar.

The first focus of chloroquine resistance in P. falciparum was described among Thai gem workers returning from nearby Cambodia in 1957 [2]. Over the subsequent 40 y, P. falciparum has developed resistance to all antimalarial drugs except the artemisinin derivatives. In 1985, Thailand became the first country to use mefloquine for the treatment of uncomplicated malaria, but resistance to mefloquine developed rapidly on both the Eastern and the Western borders [3–5]. In 1991–1992, different regimens of mefloquine with artesunate were tested in trials in Bangkok and in Karen refugee camps in Tak province [6,7]. This proved to be a highly effective and safe treatment and by 1994, when the failure rate of mefloquine monotherapy reached 60%, a combination of 25 mg/kg of mefloquine and 12 mg/kg of artesunate given over 3 d (MAS_3_) was deployed as first-line treatment in all refugee camps along the Thai-Myanmar border [8]. MAS_3_ has remained the treatment of choice since then. Very large studies have confirmed its safety and efficacy [9].

The effects of deploying the MAS_3_ combination on malaria incidence was analysed among Karen displaced people living in refugee camps in Tak province from 1986 to 1997. A 6-fold reduction in P. falciparum malaria incidence was observed, while the incidence of P. vivax malaria remained stable. This large reduction was attributed to the combination of early diagnosis (ED) by microscopy or rapid tests and prompt treatment using MAS_3_: mefloquine (25 mg/kg) and artesunate (12 mg/kg) [10].

**Figure 1:**
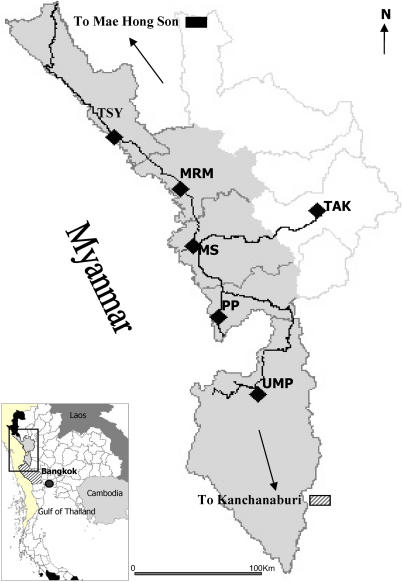
Map of Tak Province The study area is shown in grey.

In order to determine whether similar results (obtained in the well-controlled environment of camps for displaced persons) could be obtained in an open setting, a collaborative project between the Thai Ministry of Public Health, the Faculty of Tropical Medicine, Mahidol University, and the Shoklo Malaria Research Unit (SMRU, part of the Wellcome Trust–Mahidol University–Oxford Tropical Medicine Research Programme) was developed in the five border districts of Tak province (Figure 1). The Tak Malaria Initiative (TMI) aimed to provide access to ED and treatment with an artemisinin-based combination therapy (ACT) to all populations living along the northwestern border with Myanmar. We report the impact of this intervention on malaria morbidity and mortality in the targeted area by comparing epidemiological indicators before (October 1995–September 2001), during (October 2001–September 2002), and after (October 2002–September 2003) the TMI intervention.

## Methods

### Study Area and Populations

Tak province shares over 500 km of border with Myanmar and is divided into nine districts. The five districts selected for the TMI project are composed of rugged, hilly, and mostly forested terrain: Mae Ramat (MRM), Mae Sot (MS), Phob Phra (PP), Tha Song Yang (TSY), and Umphang (UMP). The remaining four districts of the province have virtually no malaria. The climate is tropical with a 6-mo rainy season (from May to early October). Mean annual rainfall varies between 1,400 mm in the southern and central areas and 2,300 mm in the northern district. The mean temperature ranges from 20.2 °C in December to 29.3 °C in April, and the annual relative humidity is above 75%.

The population at risk of acquiring malaria and targeted by the TMI project can be divided into three groups: (1) Thai citizens (approximately 300,000, of which half are Thai and the other half belong to ethnic minorities); (2) foreign nationals (FNs) (approximately 150,000, mainly migrant workers from Myanmar). The Thai National Ministry of Welfare and Social Labour provides reliable estimates of the total annual number of FNs living in the province. It includes registered workers with work permits, a mainly stable population estimated at 50,000 annually for the past 5–6 y. The families of registered workers are usually not registered but relatively stable. The remaining FNs are considered illegal, and are mostly temporary workers; and (3) displaced persons (65,000 mainly Karen displaced people living in semi-open camps in TSY, PP, and UMP (Thai Burma Border Consortium, unpublished data). Health care in the camps is provided by medical International Non-Governmental Organizations and food is supplied by a consortium of charities.

### Epidemiology of Malaria

Most of the detailed information on entomology, epidemiology, and impact of ACT deployment in this area before the TMI intervention derives from observations in the camps for displaced persons [10,11]. The area has a low and unstable malaria transmission with two seasonal peaks, in June and in December. The predominant malaria vectors are the forest-inhabiting mosquito *Anopheles dirus,* and the forest fringe mosquitoes *An. maculatus* and *An. minimus*. A preference for outdoor resting and feeding at a time when people are still active make the malaria vectors difficult to control. P. falciparum and P. vivax are the predominant species, while the two other species *(P. malariae* and *P. ovale)* are found occasionally. Malaria cases are symptomatic in all age groups [11].

### Malaria Control Programme

The Thai National Malaria Control Programme (Malaria Division) is a vertical programme and functions in the five border districts of Tak Province through a network of 37 malaria clinics. Light microscopy of Giemsa-stained thick and thin blood films are used routinely in all malaria clinics for the diagnosis of malaria. Vector control consists of indoor-chemical residual spraying (DDT until 1997, then subsequently 5% deltamethrin) once or twice a year, and impregnation of bed nets with 10% permethrin 30 mg/m^2^ every 6 mo. Patients with a malaria smear positive for P. falciparum species at the Malaria Control Programme clinics are treated with artesunate (Atlantic Company Limited) (6 mg/kg/d for 2 d) together with mefloquine (Atlantic Company Limited) (25 mg/kg) split into two doses and given 6 h apart (MAS_2_). This “shorter” regimen was shown recently to be less effective than the 3-d regimen recommended by the WHO [12,13], but was chosen on the assumption that adherence will be improved. In addition, one dose (30 mg) of primaquine (Governmental Pharmaceutical Organization) is given. Infections with *P. vivax, P. malariae,* or P. ovale are treated with chloroquine 25 mg base/kg (Governmental Pharmaceutical Organization) over 3 d, as well as primaquine (15 mg/d for 14 d). Clinically severe or complicated cases, or those with danger signs, children under 1 y of age, and pregnant women, are referred to the closest district hospital. In the study area there are one general hospital (300 beds) in MS and four community hospitals. In Thailand the private sector plays a relatively small role in malaria case management and malaria control and has not been included in this analysis.

In the Karen camps, the control of malaria is done by medical International Non-Governmental Organizations assisted by SMRU. The Malaria Division provided indoor-residual spraying in May 1998, March 2000, and April 2001. The majority of the displaced population uses bed nets (mostly non-impregnated) provided by the Thai Burma Border Consortium. The World Health Organization recommended a 3-d regimen of artesunate (4 mg/kg/d) combined with mefloquine (none on the first day of treatment, 15 mg/kg on the second day, and 10 mg/kg on the third day) is used for the treatment of uncomplicated P. falciparum infections. Its parasitological efficacy has been monitored continuously by SMRU and remains above 90% with 63 d follow-up [14]. Severe malaria cases, patients with uncomplicated hyperparasitaemia, and pregnant women are all treated in the camp clinics. Artesunate is used for severe malaria or uncomplicated hyperparasitaemia. Quinine is used for malaria in the first trimester of pregnancy. Other malaria species are treated with chloroquine 25 mg base/kg divided and given over 3 d; primaquine is not routinely used in the camps because of the lack of evidence of efficacy (except for the impractical 14-d supervised regimen) and the high incidence of G6PD deficiency (10%) in the population.

### Changes Introduced during the Tak Malaria Initiative (September 2001–October 2002)

The basis of the TMI intervention was to provide ED and treatment with an ACT to all exposed inhabitants of the five border districts. The estimated target population was 450,000 (excluding the refugee population). The people living in camps (population 65,000) already had access to ED and treatment with an ACT since 1994, so the efforts concentrated on the villages and the population of migrant workers. In August 2001, 120 medical personnel from the health centres and health posts received a refresher course in microscopic diagnosis and were trained to perform rapid diagnostic tests for malaria (using both Paracheck and Optimal). All 59 health centres and 33 health posts from the conventional health system were provided with diagnostic tools and antimalarial drugs for uncomplicated malaria. In addition, 100 malaria posts (MPs) were created in villages where health services were not readily accessible and in migrant worker settlements (Figure S1). Each MP was under the responsibility of one malaria worker, a native of the area, and usually well known and accepted by the villagers themselves. Five mobile teams were set up to reach more unstable mobile populations such as migrant workers. Rapid diagnostic tests (Paracheck-Pf and OptiMAL) were used for diagnosis in all these new structures, in combination with microscopy in health centres and mobile teams. More Paracheck-Pf tests were used during TMI because it is cheaper (US $0.6 versus US $1.5 per test), but has comparable sensitivity and specificity in the detection of *P. falciparum.* OptiMAL has the added advantage of detecting P. vivax infections, but has low sensitivity [15]. The provincial and district health offices provided the training and supervision, and SMRU performed the laboratory quality control. In addition to the 100 village workers employed in the MP, ten administrative staff were hired for data entry and programme management. By October 2001, the new structures (MP and mobile teams) represented an 80% increase in the number of malaria diagnosis and treatment facilities in the province compared to that before the implementation of TMI.

Patients presenting with symptoms of malaria were offered a rapid diagnostic test. Those with a positive test were treated immediately according to the national protocol described previously (MAS_2_).

Messages and posters advertising the location of the MPs and the identity of the MP workers were added during the second half of 2002 in all villages, and information campaigns using leaflets and radio messages in several languages (Thai, Burmese, and Karen) were conducted among villagers and migrant workers. All other activities usually run by the Malaria Control Programme (bed net distribution and indoor insecticide spraying) continued unchanged.

Despite the withdrawal of financial support for the TMI project in September 2002, all health centres continued to provide malaria diagnosis and treatment. Most of the MPs located in villages of TSY, UMP, and PP districts remained operational, offering diagnosis and treatment mainly during the two malaria peak seasons, as did the Mae Ramat mobile team.

Treatment protocols in the Karen refugee camps remained unchanged, and indoor-residual spraying with 5% deltamethrin was done once during the TMI period (March 2002), but not the year post-TMI.

### Malaria Cases Detection and Recording

Demographic characteristics, type of malaria test and its result, and treatment received were recorded for each person visiting a conventional health facility (health centres, health posts, malaria clinics), an MP in the villages, or a mobile service. Information on patients seeking care in hospitals was computerized by hospital clerks and then categorized as inpatient or outpatient. In all structures, a Thai citizen with ID card was recorded as Thai, all others were recorded as FN. A patient presenting twice for illness with the same malaria species within 1 mo was considered a treatment failure rather than a new infection because of the low incidence of malaria in the region.

Mid-year population estimates of Thai citizens by district were obtained from the provincial statistics office and provided the denominators for measuring the crude malaria incidence per 1,000 inhabitants. Malaria morbidity was represented as the annual malaria incidence per 1,000 persons and was calculated as the total number of malaria cases reported in a year (or as a yearly average of the pre-TMI period) and divided by the mid-year population of the same period. Results were recorded by malaria species and by district.

### Pregnant Women Cohort Studies

In the Karen camps (since 1986), weekly antenatal-clinic consultations have been available for all pregnant women. A thick and a thin malaria smear were done at each consultation. Annual incidence rates were calculated for P. falciparum and for P. vivax separately. Only the first episode of malaria of each species during the pregnancy was counted to calculate the number of new malaria episodes per pregnant woman-year at risk over a 1-y period.

### Cross-Sectional Surveys

To detect relative changes in the transmission of the two species, cross-sectional surveys in villages and migrant communities (Burmese workers, with or without their family, living in settlements on Thai soil) were conducted during the rainy season in 2002 using microscopy, to calculate the ratio P. falciparum/P. vivax. Survey sites were chosen in an open (nonrandomised) selection to represent each of the district characteristics (ethnic groups, distance to the health structures, transport, and known malaria transmission). The cases detected during the surveys were not included in the calculation of the annual malaria incidence.

### In Vivo Studies of Drug Efficacy

The efficacy of mefloquine and artesunate given as MAS_3_ for the treatment of patients with uncomplicated P. falciparum malaria confirmed microscopically has been regularly monitored in the area, since the introduction of this combination therapy in 1994 in the refugee camps. Briefly, patients attending the SMRU clinics and treated for P. falciparum uncomplicated malaria who gave their written consent were followed for 6 wk, daily until resolution of their parasitaemia and then weekly. Recrudescence between days 5 and 42 was differentiated from a new infection using parasite genotyping by PCR [16].

### In Vitro Studies

Antimalarial susceptibility of P. falciparum isolates has been monitored among patients recruited in camps for displaced people since 1995, and no decline in the susceptibility in vitro to mefloquine has been observed [17]. As the distribution and use of the combination of mefloquine and artesunate as MAS_2_ was considerably expanded in the province, continuous monitoring of antimalarial susceptibility to both drugs was essential. In addition to the continuous routine monitoring in the camps, in vitro monitoring using a novel double-site enzyme-linked pLDH immunodetection (commonly termed DELI) assay [17] was done on isolates from patients over 5 y old with confirmed P. falciparum and seen during the surveys. IC_50_ for artesunate and mefloquine were analysed and results compared with those from the routine monitoring.

### Entomological Surveys and Climatic Data

Entomological surveys were conducted in two Thai villages and in Maela refugee camp. Indoor catching was done in a small one-room house similar in structure to most of the Karen houses. In each site, four men (two indoors and two outdoors) collected the mosquitoes that landed on their exposed legs. Mosquito collections were done monthly, 4 d/mo in the Thai villages, and weekly, 5 d/wk in Maela camp. Mosquito species were identified on site and the heads of Anopheline mosquitoes were dissected and analysed for sporozoite carriage. The presence of P. falciparum circumsporozoite antigen, and of P. vivax antigen varieties 210 and 247, were detected by ELISA [18,19].

Monthly climatic conditions (rainfall, mean temperature, and humidity) were obtained from MS and UMP meteorological stations and from the TSY unit for the period 1996–2003. Those data provided annual trends and seasonal variations.

### Ethical Approval

The TMI project was approved by the Thai Ministry of Public Health. The cross-sectional surveys and the in vitro drug susceptibility testing were approved by the Ethical Committee of the Faculty of Tropical Medicine at Mahidol University, Bangkok. All drugs studies conducted by the SMRU have been approved by the Ethics Committee of the Faculty of Tropical Medicine, Mahidol University, the Karen Refugee Committee, and the Oxford Tropical Medicine Research Ethics Committee. Participation in all studies and surveys was entirely voluntary and participants could withdraw from the study/survey at any time.

### Statistical Analysis

Data were analysed using SPSS 10.0 software for Windows (SPSS, Chicago, Illinois, United States). Proportions were compared by *χ* ^2^ or by Fisher's exact test. Normally distributed continuous data were compared using t*-*tests and data not normally distributed by the Mann-Whitney *U* test. PCR-adjusted cure rates were calculated using Kaplan-Meier survival analysis with log rank test for significance. In vitro concentration-response data were analysed by a nonlinear regression function to determine the IC_50_ expressed in nM/l.

## Results

### Population Changes

The Thai Citizen population of the five districts increased from 258,529 in mid-year 1996 to 300,396 in 2003 with an annual population growth of 2%. The total FN population was stable and estimated at 150,000. The refugee population fluctuated because of armed attacks until October 1999, at which time only three camps remained open, and was estimated at 61,000 at mid-year 2000, increasing to 65,000 during the TMI period, and 63,400 the following year.

### Malaria Cases in the Five Districts (Thai Citizens and Foreign Nationals)

Between 1996 and 2001 (pre-TMI period), the public health services reported an average of 67,113 malaria cases per year, with little year-to-year variation. Overall, twice as many P. falciparum malaria cases were seen per year among FN than in the Thai population (mean 24,803, range 20,758–30,023 among FN versus 13,280, range 11,789–16,551 among Thais), giving an estimated annual incidence three times higher in FN than in Thais. P. vivax malaria was less common than P. falciparum in both groups, with on average 10,934 (range 8,104–12,915) cases per year in Thais versus 18,058 (range 15,588–20,573) in FNs (Table 1).

**Table 1:**
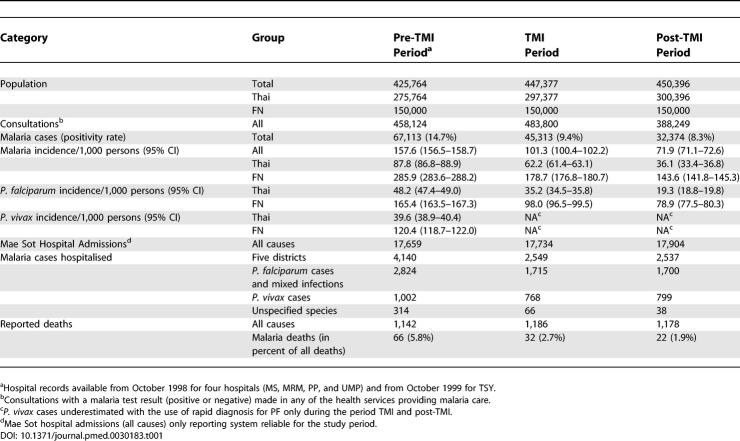
Malaria Annual Rates, Incidences, and Deaths in Thai and FN Populations

During the TMI period (October 2001–September 2002), the public services diagnosed 45,313 confirmed malaria cases, of which 25,159 were P. falciparum malaria cases, i.e., a 34% (95% CI, 33.5–34.4) reduction when compared to the pre-TMI period. The reduction was seen in both Thais and FN: from 13,280 to 10,460 cases (a reduction of 21.2%; 95% CI, 20.5–21.9) and from 24,803 to 14,699 cases (a reduction of 40.7%; 95% CI, 40.1–41.4), respectively.

In the year following the intervention (October 2002-September 2003), there was a further 29.9% (95% CI, 29.4–30.5) overall reduction in P. falciparum cases. This was significantly greater in Thais than in FN: from 10,460 to 5,794 cases (a reduction of 44.6%; 95% CI, 43.7–45.6) and from 14,699 to 11,834 (a reduction of 19.5%; 95% CI, 18.9–20.1) respectively (*p* < 0.001) and was reflected in the decrease in the estimated malaria incidence (Table 1).

P. vivax malaria cases during and following the TMI period could not be compared accurately, because the Paracheck rapid test only detects P. falciparum.

The number of hospital admissions of all causes was reliably recorded only in Mae Sot hospital and was stable throughout the three time-periods. By contrast, there was a marked decrease in the number of admissions due to malaria (Table 1). The number of hospitalised cases continued to decrease during the year post-TMI in all but two district hospitals (TSY and MRM), where it increased substantially, due to an influx of imported cases from Myanmar. Likewise, the total number of hospital deaths from all causes was stable but there was a 51.5% (95% CI, 39.0–63.9) reduction in malaria-related deaths (Table 1).

In the Karen displaced population a significant decrease in malaria incidence also occurred, although it was less marked than that observed in the Thai and FN populations. In the pregnant women cohort, the incidence of P. falciparum and P. vivax remained low and stable (Table 2). Indeed, since 1997 the falciparum malaria incidence in this cohort has remained below 0.2 episodes per pregnant-woman-year, after having fallen from 2.60 in 1986 to 1.28 in 1992 and 0.37 in 1996. This decrease is a direct consequence of the overall reduction in transmission of P. falciparum in the camps population following the deployment of MAS_3_ [10].

**Table 2:**
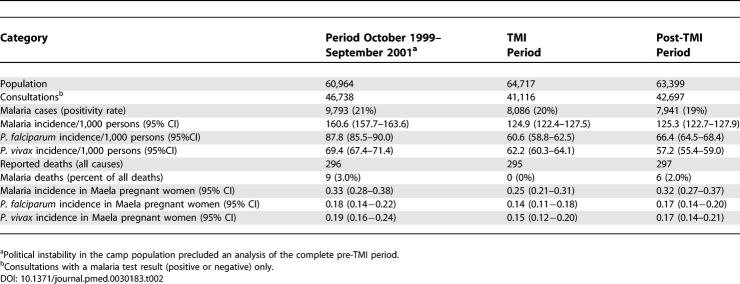
Malaria Annual Rates, Incidences, and Deaths in Karen-Displaced Populations

In all three population groups, P. falciparum malaria was seen at all ages but with a greater predominance of adult males among patients in the FNs and the refugees than in the Thai citizen population (Table 3).

**Table 3:**
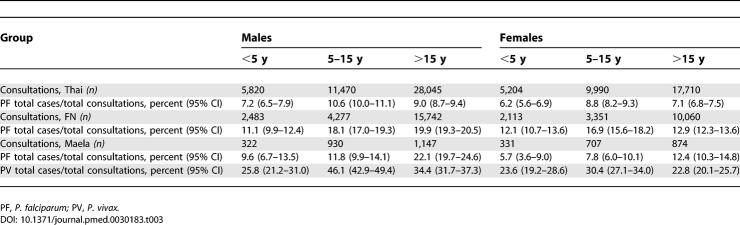
Distribution of Malaria Cases by Age and Sex, in Thais, FN, and in Maela Camp (TMI Period)

### Malaria Prevalence in Cross-Sectional Surveys

Between June and October 2002, 43 sites were surveyed. Overall participation was 80% of the expected population of villagers and 90% of the FN communities. An additional 760 Burmese persons living in Burmese villages opposite four of the screening sites were included in the results, and are further referred to as Burmese villagers. The prevalence and ratios of P. falciparum and P. vivax in each population category are presented in Table 4. While P. falciparum was the most common species before the intervention (Table 1), P. vivax became the predominant species among villagers and FN targeted by TMI. This translated into a change in the P. falciparum to P. vivax ratio from 1.3 prior to TMI to less than 1 during TMI, while P. falciparum remained predominant among Burmese villagers where no intervention took place (Table 4). The higher prevalence of parasitaemia in adults was evident in the FN and the Burmese villagers, and the percentage of gametocyte carriers was also significantly higher among Burmese villagers compared to migrants and Thai villagers (*p* < 0.001). Finally, there was a clear relation between the prevalence rates and the distance to the Myanmar border, with the highest prevalence rates found in migrant communities closest to the border or in Burmese villagers.

**Table 4:**
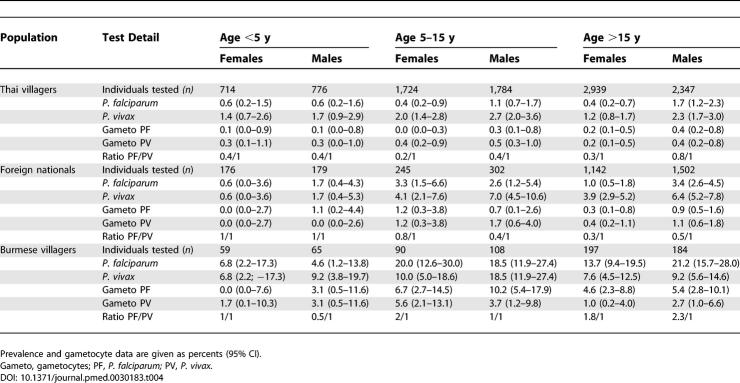
P. falciparum and P. vivax Prevalence and Their Ratios, and Gametocyte Prevalence within the Three Population Categories, by Sex and Age Groups

### Entomological and Climatic Data

The annual mean humidity, temperature, and rainfall did not change significantly in the area during the study period (October 1995–September 2003). Surveys in the villages took place between September 2001 and August 2002, and in Maela camp between June 2002 and February 2003.

*Anopheles minimus, A. maculatus,* and A. dirus were the three main vectors collected in both villages and in the camp. *Anopheles* abundance remained unchanged in Maela camp compared to previous surveys [10], and species were in similar proportions. In the villages, A. minimus was the predominant species (80%), with two high peaks, one in the early rainy season and the other in the early cold season.

Between October 2002 and February 2003, 17,246 mosquitoes collected in Maela camp and 5,575 caught in the villages were analysed by ELISA to detect sporozoite carriage. Sporozoite rates were low in all sites (0.21% in Thai villages and 0.23% in Maela camp for *P. falciparum,* 0.12% and 0.24% respectively for P. vivax). The annual entomological inoculation rate derived from these results was 0.087 (95% CI, 0.069–0.105) infective bites per person per year for P. falciparum and 0.092 (95% CI, 0.073–0.118] per year for P. vivax.

### In Vivo Studies of Drug Efficacy and In Vitro Susceptibility

Efficacy of the combination of mefloquine and artesunate (as MAS_3_) prior to the TMI period was 96.2% (95% CI, 93.6–97.8). During the TMI period, the cure rate assessed at 42 d in 79 patients was also 96.2% (95% CI, 89.4–98.7) and remained unchanged the year after the TMI period, at 96.1%, (95% CI, 93.4–98.8), in a study involving 210 patients.

**Figure 2:**
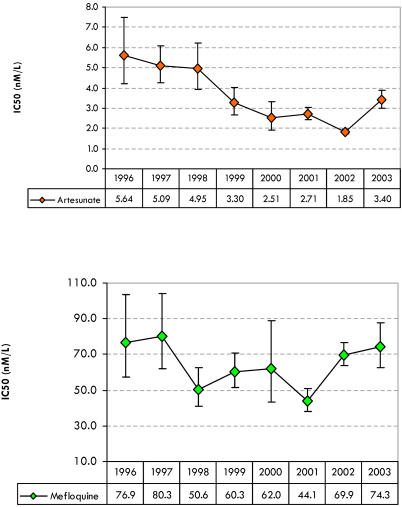
Changes in Drug Sensitivity of P. falciparum Isolates Isolates from primary infections were collected at SMRU clinics between 1996 and 2003 and assayed for sensitivity to artesunate and mefloquine, IC_50_ geometric means given as nM/l (95% CI).

Temporal trends in the geometric mean IC_50_ (nM/l) for mefloquine and artesunate in isolates from primary P. falciparum infections treated at SMRU clinics are shown in Figure 2 for the period (1996–2003). There was a significant increase in the in vitro susceptibility over time for artesunate (Pearson's correlation coefficient *r* = −0.404; *p* < 0.001). A 42% (95% CI, 33.0–53.0) increase in susceptibility was also observed for mefloquine between 1996 and 2001, but by 2003 IC_50_ levels returned to those described in 1996. The mean IC_50_ for mefloquine and artesunate tested by the DELI assay in the isolates obtained during the prevalence surveys in 2002 were not significantly different from those treated in SMRU clinics over the same period using the isotopic microtest: artesunate IC_50_ given as geometric mean in nM/l (95% CI) was 1.80 (1.50–2.09) with DELI versus 1.85 (1.72–1.96) with microtest (*p* > 0.05); mefloquine IC_50_ was 59.06 (44.12–78.84) versus 69.92 (63.65–76.43) (*p* > 0.05).

## Discussion

The beneficial effects of the strategy of ED and the 3-d regimen of ACT implemented in 1994 in the Karen refugee camps and described previously, have persisted for over 10 y. The incidence of P. falciparum malaria cases has decreased continuously in all refugee camps, including those situated in adjacent provinces, with a concomitant reduction in P. falciparum-related morbidity and mortality. This was particularly evident in the less mobile segments of the population, e.g., pregnant women.

In this project, we assessed the implementation of the same strategy of rapid diagnosis and treatment with ACT in a much (seven times) larger population living adjacent to the Burmese border in Tak province.

During the TMI project, the overall number of consultations increased only slightly despite an increased diagnostic capacity, probably because of the transfer of consultations from the malaria clinics to the newly created village-based MPs. Changes in diagnostic tools from microscopy to a rapid test (that detects P. falciparum only) underestimated the number of P. vivax cases. Nevertheless, the reduction by one-third in the total number of falciparum malaria cases observed in the Thai population during the TMI project was sustained a year later, and these results are consistent with a reduction in the number of hospital admissions for P. falciparum. This decrease in falciparum malaria is further confirmed by the results obtained during the surveys in the villages, showing a change to predominance of P. vivax species and very low rates of gametocyte carriage. The predominance of P. vivax malaria, the changes in P. falciparum prevalence, and low gametocyte rates confirm the reduction of P. falciparum burden in this population. The overall reduction in falciparum malaria cases in the Thai population (a reduction of 56.4%; 95% CI, 55.5–57.2) was more marked than that observed in FN, and is probably explained by better access and coverage for diagnosis and treatment in the Thai population. Indeed, the proximity of a large reservoir of malaria across the border in Burma and considerable cross-border population movement presumably explain why the impact of this initiative was less than observed previously in relatively contained refugee camps. These factors also explain the plateau observed in the number of hospital cases during the post-TMI period, as a result of an increase in cross-border cases in two of the five districts (TSY and MRM). In order to obtain the desired impact, coverage must be sufficient. In this project, 80% of all villages had access to a rapid diagnosis and prompt treatment with ACT, while in the FN population this proportion was closer to 50%. Improved malaria control in the adjacent region will be needed to reduce these figures much further. An additional factor, which may have limited the impact, is the use of a 2-d artesunate regimen in combination with mefloquine (i.e., MAS_2_). This treatment has a lower efficacy than the generally recommended 3-d regimen, and concerns have been expressed about the higher mefloquine pressure it exerts on the parasite populations [20].

In the refugee camp population, where the ED and treatment with ACT were deployed in 1994, we expected the impact of the TMI intervention to be less noticeable. Indeed, the rate of infective bites per person for P. falciparum is now as low as one every 10 y. But movement of people in and out from the camp perimeter (where transmission is higher) explains why patients are still presenting with malaria in the camp clinics and indicates that transmission is not completely interrupted, especially in the forested fringes where vectors are more abundant.

The large-scale deployment of the MAS has not modified the efficacy of the treatment, which remains extremely high, and there is no in vitro change in susceptibility to either drug, although there was a shift of the mefloquine IC_50_ in 2003 back to the values of 1996. This is of concern and could be related to the use of unprotected mefloquine in the area, as well as to the less effective regimen (using 2 d of artesunate, MAS_2_) deployed by the Thai Ministry of Health [20,21].

During the 2 y of this project we were unable to collect information on adverse effects of the treatment. However we have conducted studies in thousands of patients in this area and consistently found that the MAS_3_ regimen was well tolerated [22]. We had no reports of patient complaints regarding treatment. Indeed, the rapidity of action following antimalarial treatment with concomitant reduction of symptoms was generally recognised by the population and encouraged treatment-seeking behaviour.

The decrease in malaria morbidity observed during, and sustained 1 y after the TMI project, the persistence of high cure rates of the combination therapy 10 y after its first introduction in the province, the persistently low sporozoite rate, and the rapid acceptance of newly established MPs, all indicate that it is feasible to extend early malaria diagnosis and provide adequate treatment even to remote communities. The impact of this intervention was seen rapidly (within a year of introduction) in this area of low and seasonal transmission. We were not in a position to perform a cost-benefit analysis of the TMI project, but the public health benefits were evident given the large reductions in morbidity and mortality observed. These results give further support to the large-scale use of ED and prompt treatment with ACT, a strategy that has shown similar results in KwaZulu-Natal [23] and has now been recommended in all endemic regions to circumvent the spread of P. falciparum drug resistance and to reduce the impact of malaria [13].

## Supporting Information

Alternative Language Abstract S1 Translation of Abstract into French (22 KB DOC)

Figure S1 Picture of a Malaria Post in Tak Province (189 KB JPEG)

**Editors' Summary**

**Background.**

Malaria kills about a million people worldwide every year. Most of these deaths are in children. One of the most serious problems in the battle against malaria is that the parasites that cause the disease are able to change and become resistant to the drugs used to treat it. Widespread drug resistance in the parasite that causes the most serious type of malaria, *Plasmodium falciparum,* is of particular concern. Although Africa is the part of the world with the greatest number of malaria cases, the problem of drug resistance is most severe in Southeast Asia; the parasite even has some resistance to drugs introduced just a few years ago. A new drug, artemisinin, is now the most effective treatment available. So far, there seems to be no resistance to artemisinin. It is recommended that artemisinin should be given as a pill that also contains one of the older drugs. It is thought that this approach will both slow down the development of resistance to the older drug and delay the appearance of artemisinin resistance. Several research projects have shown encouraging results with this combined form of drug treatment.

Along the Thai-Burmese border there are a number of camps for displaced people (refugees). In a malaria programme for people in the camps, efforts were made to diagnose cases early, and people found to have malaria were then treated with a combination pill, containing artesunate (a derivative of artemisinin) and another drug, mefloquine. A study of this programme found that it made a big reduction in the number of cases of the most serious form of malaria, and suppressed the advance of resistance to mefloquine.

**Why Was This Study Done?**

It was decided to introduce the same malaria strategy for all the estimated 450,000 people living in five border districts of the remote Tak province in northwestern Thailand. It was necessary for the impact of this larger programme to be studied to see whether this new approach to malaria control was effective in a remote area—not just in the particular circumstances of the camps, but on a wider scale.

**What Did the Researchers Do and Find?**

Village volunteers were trained to identify cases early, using rapid diagnostic tests. Health facilities were improved and increased in number, so that the combined treatment could be given to those who needed it. The researchers conducted surveys of malaria cases before, during, and after the programme. They recorded the type of malaria test used and the result, and the treatment each person received, whether they were treated at health centres, at village health posts, or by mobile teams. They found that cases of malaria caused by P. falciparum fell by around a third. There was no change in the number of cases caused by the other malaria parasite common in the area, P. vivax. The malaria death rate was halved.

**What Do These Findings Mean?**

The reduction in the number of deaths shows that the new approach to treatment is more effective, and the drop in the number of cases shows that the transmission of malaria was also reduced. This means that, even in a remote area, a programme that involves both improved diagnosis and the combined artesunate-mefloquine treatment can have major benefits for the people living there. This is important not just for Thailand but also for other parts of the world where malaria is a problem.

**Additional Information.**

Please access these Web sites via the online version of this summary at http://dx.doi.org/10.1371/journal.pmed.0030183.

• The Global Malaria Programme of the World Health Organization has a Web site with information about the disease and the global efforts to fight it; the site also has links to other organizations and to useful publications

• The US Centers for Disease Control and Prevention has Web pages on malaria

• MedlinePlus brings together authoritative information from the US National Library of Medicine, National Institutes of Health, and other government agencies and health-related organizations; MedlinePlus has information specifically on malaria

• Thailand's Ministry of Public Health has a Web site that includes information on malaria and malaria control in the country

• The organization that spearheads the research described in this article is the Shoklo Malaria Research Unit

## Abbreviations

ACT: artemisinin-based combination therapy
CI: confidence interval
DELI: double-site enzyme-linked pLDH immunodetection
ED: early diagnosis
FN: foreign national
IC_50_: 50% inhibitory concentration
MAS: mefloquine-artesunate combination therapy
MAS_number_: [number]-d MAS
MP: malaria post
MRM: Mae Ramat district
MS: Mae Sot district
PP: Phob Phra district
SMRU: Shoklo Malaria Research Unit
TMI: Tak Malaria Initiative
TSY: Tha Song Yang district
UMP: Umphang district
