# Supplementary material for: Deployment of Early Diagnosis and Mefloquine- Artesunate Treatment of Falciparum Malaria in Thailand: The Tak Malaria Initiative
Source: PLoS Med. 2006 Jun 6;3(6):e183. doi: 10.1371/journal.pmed.0030183 (PMC1470664; doi:10.1371/journal.pmed.0030183)
Supplement: Supplementary file 1 [file pmed.0030183.sd001.doc]

**Translation of the abstract of “Large Scale Deployment of Early Diagnosis and Mefloquine-Artesunate Treatment of Falciparum Malaria on the North-Western Border of Thailand: the Tak Malaria Initiative” in French by V. I. Carrara.**

**Traitement antipaludique de méfloquine et d’artésunate associé à un diagnostic précoce de *P. falciparum* à la frontière entre la Thaïlande et la Birmanie: l’impact de cette stratégie déployée dans le cadre du projet “Tak Malaria Initiative”.**

**Titre abrégé: diagnostic précoce et association antipaludique contenant un dérivé de l’artémisinine (ACT) en Thaïlande**

**Résumé**

**Introduction :** Un diagnostic antipaludique précoce et un traitement de trois jours associant la méfloquine à de l’artésunate (MAS_3_) ont réduit de manière importante la transmission du paludisme à *P. falciparum* et empêché la progression de la résistance à la méfloquine dans les camps de réfugiés situés à la frontière entre la Thaïlande et la Birmanie, dans une zone de basse endémie où s’est développée une résistance à tous les antipaludiques autres que les dérivés de l’artémisinine. Cette même stratégie a été offerte aux 450,000 habitants des cinq districts de la province de Tak adjacents à la frontière birmane, dans le cadre du projet « Tak Malaria Initiative » (TMI).

**Méthode :** Des postes de santé ont été créés dans les villages pour renforcer le service sanitaire en place. Des auxiliaires de santé ont été formés à la lecture des tests rapides de diagnostic antipaludique, ainsi qu’à l’utilisation de l’association méfloquine et artésunate. Les cas, les hospitalisations et les décès dus au paludisme, enregistrés pendant les six années précédant le projet TMI, durant le projet et l’année suivante, ont été analysés, de même que les résultats des études transversales conduites avant et pendant le projet TMI.

**Résultats :** Les cas confirmés de paludisme à *P. falciparum* durant le projet TMI ont diminué de 34% [95% IC : 33.5 ; 34.4] et les hospitalisations de 39% [95% IC : 37.0 ; 39.9], alors que le nombre de patients hospitalisés en raison d’un paludisme à *P. vivax* n’a pas varié. Les décès causés par le paludisme (32 durant le projet et 22 l’année suivante) ont diminué de 51.5% [95% IC : 39.0 ; 63.9] par rapport aux années précédentes. Les résultats des études transversales montrent que *P. vivax* est devenue l’espèce prédominante dans les villages thaïlandais, ce qui n’est pas le cas des villages situés de l’autre côté de la frontière. La suppression de la transmission du plasmodium à *P. falciparum* dans les populations de réfugiés continue, son incidence reste basse et l’association de méfloquine et d’artésunate est toujours extrêmement efficace *in vivo*.

**Conclusion :** La formation d’auxiliaires de santé à la lecture des tests rapides de diagnostic antipaludique, ainsi qu’à l’utilisation de l’association méfloquine et artésunate a permis d’obtenir, grâce à un diagnostic précoce, une réduction de la morbidité et de la mortalité associées au *P. falciparum* dans des endroits reculés situés le long de la frontière entre la Thaïlande et la Birmanie.
